# Questionnaire survey of working relationships between nurses and doctors in University Teaching Hospitals in Southern Nigeria

**DOI:** 10.1186/1472-6955-5-2

**Published:** 2006-02-21

**Authors:** Roseline I Ogbimi, Clement A Adebamowo

**Affiliations:** 1 Institute Of Public Administration and Extension Services, University of Benin, Benin-City, Nigeria; 2 West African Bioethics Training Program, Division of Oncology, Department of Surgery, College of Medicine, University of Ibadan, University College Hospital, Ibadan, Oyo State, Nigeria

## Abstract

**Background:**

Smooth working relationships between nurses and doctors are necessary for efficient health care delivery. However, previous studies have shown that this is often absent with negative impact on the quality of health care delivery. In 2002, we studied factors that affect nurse-doctor working relationships in University Teaching Hospitals (UTH) in Southern Nigeria in order to characterize it and identify managerial and training needs that might be used to improve it.

**Method:**

Questionnaire survey of doctors and nurses working in four UTH in Southern Nigeria was done in 2002. The setting and subjects were selected by random sampling procedures. Information on factors in domains of work, union activities, personnel and hospital management were studied using closed and open-ended questionnaires.

**Results:**

Nurse-doctor working relationships were statistically significantly affected by poor after-work social interaction, staff shortages, activist unionism, disregard for one's profession, and hospital management and government policies. In general, nurses had better opinion of doctors' work than doctors had about nurses' work.

**Conclusion:**

Working relationships between doctors and nurses need to be improved through improved training and better working conditions, creation of better working environment, use of alternative methods of conflict resolution and balanced hospital management and government policies. This will improve the retention of staff, job satisfaction and efficiency of health care delivery in Nigeria.

## Background

Smooth working relationships between doctors and nurses are prerequisite for efficient delivery of health care. This has often been overlooked to the detriment of patients' care and increased cost to the health care system, particularly in developing countries. In many countries, doctors determine the scope of nursing practice and education, and can directly define the limits of nursing knowledge[1,2]. In Nigeria, doctors also head public health care institutions which gives them additional opportunities to influence the training of nurses [3]. Nevertheless, several authors have argued that these working relationships are changing and should be examined against prevailing developments in the professions, society and workplace [4-6].

Gjerberg and Kjolsrod[7] opined that increasing male entry into nursing and female entry into medicine may change the perception of the role of gender in doctors-nurses working relationships. In many countries, including Nigeria, nursing is moving away from the traditional practice-based training towards dynamic university based education. Furthermore, nursing education is increasingly socialized and this may ensure that nurses play a more independent professional role[8]. Older nurses may also expect traditional cultural respect due to an older person from often relatively younger doctors[7,9]. With these developments, nurses and other professionals in the health care industry are challenging the subordination of their occupational status to that of physicians[10]; nevertheless some authors have warned that higher status workers could just as likely be victimized as those in lower status[11].

In Nigeria, the working relationships between doctors and nurses have also been affected by episodes of withdrawal of services by both doctors and nurses in recent times. This has occurred within the context of changing political and social environment, crippling economic difficulties associated with agitations by labor unions and civil society. These factors also affected the health care industry and relationships between various categories of health workers. Inter-professional conflicts in the Nigerian health care delivery system has been described as very intense, deep-rooted and crippling [12,13]. There is no previous study of the factors that influence nurse-doctor working relationships in Nigeria, therefore this study was conducted in order to identify such factors and the changes that are needed in order to improve these relationships and enhance delivery of better and more efficient health care

## Methods

There were nine University Teaching Hospitals (UTHs) in the southern health zone of Nigeria in 2002[14] when this study was conducted. Four of them were selected by simple balloting. Three of these, located in Cross-River, Edo and Osun States, were established over 2 decades ago while the fourth located in Anambra State was just over a decade old. In total, there were 842 doctors and 1532 nurses in these hospitals. We obtained approval from the management of each of them to conduct a survey of their staff. Using the list of nurses and doctors in each hospital as sampling frames, 50 nurses and 25 doctors were selected from each hospital by systematic sampling to give a total of 100 doctors and 200 nurses for this study.

A self administered survey instrument was developed from the literature and informal discussions with healthcare workers. It was pre-tested and modified accordingly. The first sets of questions in the survey instrument elicited information on the demographic characteristics of the respondents. Other questions were categorized into personal, union and work activities, hospital management issues and how these affect nurse-doctor working relationships. In open-ended questions, respondents were asked to indicate other issues that they think may affect nurse-doctor working relationships. The responses were coded using the variables in the responses to determine the coding guide. The open-ended questions were coded and quantified.

Factors affecting nurse-doctor relationships suggested to the respondents included 'cultural demands' of respect from the younger generation, informal relationships, inadequate development of interpersonal skills, personal characteristics and refusal to take advice. Occupational group factors suggested include disregard for one's profession, contentious occupational union activities, type of professional training and the wish to work without a doctor or a nurse. Factors related to patients' care such as, provision of insufficient information about patients' diagnosis, lack of adequate attention to patients, uncooperative work attitudes, inadequate drug administration, poor attitude to work, refusal to come for duty calls, interference, negligence of duty and staffing insufficiency were assessed. Government and hospital management factors that were assessed included unfavorable management decisions such as the category of health care worker who can head UTHs.

Respondents had options of "strongly agree" scored as 5; "agree" scored as 4; "undecided" scored as 3; "disagree" scored as 2 and "strongly disagree" scored as 1 on a five point Likert scale. The content validity was determined by giving the questionnaire to consultants in health care organizations' management to check whether it will test what it is meant to test and from literature[3,7,15],after which the questionnaire was pre-tested among health care workers who were not participants in the study. Chi-square test was used to determine whether differences in responses between nurses and doctors were statistically significant. The level of significance was fixed at 0.05. Missing data on each item were considered as non-response and these were not included in the chi-square calculations. For multivariate analysis in order to evaluate the agreement between nurses and doctors on the impact of factors of interest on the nurse-doctor working relationship, items with multiple response levels were collapsed into binomial variables of "having effect" and "having no effect". Logistic regression models of the dependent variable on the predictors were run and multivariate p-values are reported.

## Results

Most of the doctors (n = 67, 81.0%) and nurses (n = 158, 79.5%) returned the questionnaire. Overall there were more females (61.3%) than males (36.0%) in this study; 6 participants did not indicate their sex. The age of the respondents ranged from 27 to 60 years. The nurses in this study were on the average older than the doctors (mean age [SD] 44.3 [7.0] vs. 35.7 [5.6] years. t-test = 9.55, p-value < 0.001). Among nurses, 84.3% were females compared to 13.6% of the doctors. Most, 80%, of the respondents were married. Among the nurses 87.7% were married compared to 66.7% of the doctors (Table 1).

**Table 1:** Baseline Characteristics of Sample of doctors and nurses surveyed in University Teaching Hospitals in Southern Health Zone of Nigeria, 2002

**Variables**	Doctors* (N = 67)		Nurses* (N = 158)	
	**N (%)**	**Mean (SD)**	**N (%)**	**Mean (SD)**
**Age**	65	35.7 (5.6)	154	44.3 (7.0)
**Sex**				
Male	57 (85.1)		24 (15.2)	
Female	9 (13.4)		129 (82.7)	
Missing	1 (1.5)		5 (3.2)	
**Marital Status**	75		157	
Married	44 (65.7)		136 (86.1)	
Unmarried	23 (34.3)		22 (13.9)	

Totals may be less than N on account of missing data

There was no significant difference between doctors (92.5%) and nurses (82.9%) who considered the working relationship between nurses and doctors as cordial (p-value = 0.16). Doctors (66.7%) are more likely than nurses (57.5%) to suggest that inadequate development of interpersonal skill play a role in their working relationship but this was not significant in multivariate analysis adjusted for age and sex (multivariate p-value adjusted for age and sex [MV p-value] = 1.00). More nurses (52.1%) compared to doctors (24.2%) think that poor social interaction outside work influences their working relationships and this remained statistically significant after adjusting for age and sex (MV p-value = 0.002). Other potential personal factors contributing to the working relationships such as perception of respect (MV p-value = 0.24), compliance with advice (MV p-value = 0.52), personality traits (MV p-value = 0.30) and communication gaps (MV p-value = 0.28) while different between the two groups, did not reach statistical significance (Table 2). From the open-ended questions, nurses commonly noted that they were often not promptly notified about patients with infectious diseases such as HIV/AIDS thereby delaying the deployment of barrier nursing techniques. On the other hand, doctors often stated that nurses delayed reporting conditions such as post-operative anuria.

**Table 2:** Personal Factors Perceived by Respondents as Affecting Nurse-Doctor Working Relationship in University Teaching Hospitals in Southern Health Zone of Nigeria, 2002

**Personal Factors**	**Doctors (N = 67)**		**Nurses (N = 158)**		**Multivariate p-value**
	
	**Effect N (%)**	**No effect N (%)**	**Effect N (%)**	**No effect N (%)**	
Interpersonal skills	44 (66.7)	22 (33.3)	88 (57.5)	65 (42.5)	1.00
Social interaction outside work	15 (24.2)	47 (75.8)	73 (52.1)	67 (47.9)	0.002
Perception of being respected	37 (56.9)	28 (43.1)	94 (66.2)	48 (33.8)	0.24
Compliance with advice	45 (67.2)	22 (32.8)	99 (65.6)	52 (34.4)	0.52
Personality traits	39 (59.1)	27 (40.9)	96 (65.3)	51 (34.7)	0.30
There is communication gap between me and the doctor/nurse	25 (37.9)	41 (62.1)	77 (50.7)	75 (49.3)	0.28

With respect to work related factors that affect doctor-nurse working relationships, nurses (79.5%) were more likely than doctors (59.4%) to complain that staff shortage (MV p-value = 0.004) is a significant cause of poor doctor-nurse working relationships. Other work related factors such as inadequate drug administration (MV p-value = 0.12), dictating how work should be done (MV p-value = 0.13), provision of inadequate information (MV p-value = 0.28), poor work attitude (MV p-value = 0.76), failure to respond to call duty (MV p-value = 0.45), inadequate attention to patients (MV p-value = 0.51), uncooperative attitude at work (MV p-value = 0.99) and negligence of duty (MV p-value = 0.06) were not significant predictors of doctor-nurse working relationships (Table 3). In general, more nurses (53.3%) than doctors (33.8%) had a good opinion of the attitude of the other group to work, but this was not statistically significant (p=.07) (Table 3).

**Table 3:** Work Activity Factors Perceived by Respondents as Affecting Nurse-Doctor Working Relationship in University Teaching Hospitals in Southern Health Zone of Nigeria, 2002

**Work Activity Factors**	**Doctors (N = 67)**		**Nurses (N = 158)**		**Multivariate p-value**
	
	**Effect N (%)**	**No effect N (%)**	**Effect N (%)**	**No effect N (%)**	
Staff shortage	38 (59.4)	26 (40.6)	120 (79.5)	31 (20.5)	0.004
Inadequate drug administration	50 (76.9)	15 (23.1)	86 (57.7)	63 (42.3)	0.12
Dictating how work should be done	45 (69.2)	20 (30.8)	117 (79.6)	30 (20.4)	0.13
Amount of information provided about patients	45 (69.2)	20 (30.8)	97 (65.5)	51 (34.5)	0.28
Attitude to work	40 (67.8)	19 (32.2)	82 (62.1)	50 (37.97)	0.76
Response to call duty	37 (61.7)	23 (38.3)	106 (73.6)	38 (26.4)	0.45
Uncooperative attitude at work	42 (66.7)	21 (33.3)	100 (69.9)	43 (30.1)	0.99
Negligence of duty	49 (76.6)	15 (23.4)	92 (65.3)	49 (34.8)	0.06

Nurses (67.5%), more than doctors (22.7%), felt that the union activities of the other profession were inimical to the interests of their profession (MV p-value <0.001) and 77.5% of nurses compared to 54.1% of doctors felt that there was often disregard for their profession by the other group (MV p-value = 0.02). While majority of the nurses and doctors understand that they need each other for effective health care delivery, more nurses (32.5%) than doctors (13.9%) wish they could complete their work without the other (MV p-value = 0.006). Few of the respondents ascribed nurse-doctor relationships to the type of professional training they received (MV p-value = 0.14). More nurses (74.2%) than doctors (7.7%) considered the policy of the government (MV p-value < 0.001) and that of hospitals' management (nurses 54.5% vs. 6.6% of doctors MV p-value < 0.001) to be inimical to their professional interests (Table 4). Majority of nurses (86.1%) compared to doctors (29.2%) want the headship of hospitals open to election by all health care professional groups in the hospital.

**Table 4:** Occupational Group and Hospital Management Factors Perceived by Respondents as Affecting Nurse-Doctor Working Relationship in University Teaching Hospitals in Southern Health Zone of Nigeria, 2002

**Occupational Group and Hospital Management Factors**	**Doctors (N = 67)**		**Nurses (N = 158)**		**Multivariate p-value**
	
	**Effect N (%)**	**No effect N (%)**	**Effect N (%)**	**No effect N (%)**	
Occupational union activities	15 (22.7)	51 (77.3)	104 (67.5)	50 (32.5)	< 0.001
Disregard for profession	33 (54.1)	28 (45.9)	110 (77.5)	32 (22.5)	0.02
Type of professional training received	22 (33.4)	43 (66.2)	72 (46.8)	82 (53.3)	0.14
Government policy	5 (7.7)	60 (92.3)	115 (74.2)	40 (25.8)	< 0.001
Hospital Management	4 (6.1)	62 (93.9)	85 (54.5)	71 (45.5)	< 0.001

## Discussion

It is reassuring to note that majority of doctors and nurses in Nigeria considered the working relationships between the two professions to be cordial, but problems remain. In this study, we found that, proportionally, there were more female nurses than female doctors. Given the role that gender perception plays in doctors-nurses working relationships[7], we opine that it is necessary to increase the recruitment of men into nursing and women into medicine in order to balance the gender distribution, reduce gender-role-perception based conflicts and enhance nurses-doctors working relationships.

Bad behavior among both doctors and nurses has been linked to poor retention of staff in the health care system and poor clinical outcomes[16,17]. While some authors think doctors are the major sources of these conflicts[18], others have blamed medical training programs that set up a hierarchical model with nurses in a relatively subservient role[16]. In the opinion of Witz[19], doctors' behaviors serve as vital demarcation strategies to confirm physicians' autonomy in inter-occupational relationships with nurses. In our study, factors such as inadequate development of interpersonal skills, perception of respect, compliance with advice, personality traits and communication gaps were more commonly reported by nurses than by doctors as having an effect on nurses-doctors working relationships, although these did not reach statistical significance. Nevertheless many more nurses than doctors wished that they could do their work without the other professional group.

Staff shortage was an important determinant of poor nurses-doctors working relationships in our study. This is consistent with findings of other studies that showed that this factor also plays an important role in patients' outcome [17,20]. Perennial staff shortage is common in health care institutions in developing countries, including Nigeria, due to decades of economic depression and lack of development[21]. This situation has been worsened in recent times by the recruitment of health care workers in developing countries by developed countries[22]. Inadequate staff leads to inefficient health care delivery, perceptions of uncooperative work attitude between health care professionals and further inefficiencies in health care delivery. This may increase the risk of disruptive behavior among health care workers which sets off a feedback mechanism where staffing shortage increases tension in the working environment leading to further exodus of health care workers[16].

Another major factor influencing the working relationships between nurses and doctors in our environment was the union activities of professional groups. We found that nurses more than doctors felt that the union activities of the other professional group were inimical to the professional interests of their group. One of the major responses to decades of poor government and economic depression in developing countries has been the radicalization of workers' unions. Withdrawal of services became a frequent tool for negotiating new working conditions and display of grievances about government policies. Such activities tended to polarize workers, particularly in a multidisciplinary environment like health care, where some groups, usually doctors, may be considered more privileged than others[23]. With return of more stable democratic government (since 1999 in Nigeria) and better labor relationships, the impact of this factor is likely to diminish in future.

Peter reports lack of appreciation of nursing knowledge by physicians and others[20]. Our study also shows that there was perception of lack of appreciation of the knowledge of the other professional group by both nurses and doctors, but this was more prevalent among doctors than nurses[16,17]. Furthermore, more nurses than doctors wanted the post of the chief executive of hospitals to be open to all professionals in the health care system, in the belief that this will positively influence the conditions of service of health care workers and their sense of belonging. Other health care professionals in Nigeria consider government policies such as those related to the headship  of public health care institutions discriminatory[13]. According to Ogbimi[25], occupational prestige is determined by its sophistication, effectiveness, exclusiveness and accessibility of service to the public. The current situation where headship of hospitals is the sole preserve of doctors arose after series of protracted doctors' withdrawal of services and may account for the overwhelmingly positive response by doctors to government and hospital management policy compared to that of nurses in this study. We also found that the degree of social interaction between nurses and doctors outside the working environment was a predictor of nurse-doctor working relationship, but this may be a reflection of the Nigerian social and cultural structures that are not necessarily generalizable.

Our findings should be interpreted within the context of the limited nature of the development of the instrument used. A more comprehensive sampling of all the doctors and nurses in the region covered by the study would have yielded more information. In addition, we did not keep institution specific information hence could not adjust for the different institutions in the analysis. In addition, responses were voluntary and may have been drawn largely from respondents interested in this issue.

## Conclusion

Our study identified staff shortage, lack of appreciation, particularly of nurses' work by doctors, activist unionism and government policies that were perceived to be more favorable to doctors as inimical to good working relationships between nurses and doctors in Nigeria. This significantly contributes to poor health care delivery and reduced efficiency of the health care system – problems that the traditionally weak health care system of a developing country like Nigeria can ill afford. Health care managers and aid agencies that partner with developing countries need to urgently consider measures to combat this problem.

More training and improvement in nurses' working conditions will ameliorate the nursing staff shortage and lead to better and more efficient health care delivery, improved patient outcomes, less morbidity and mortality, reduced hospital stay and substantial cost savings. Investment in nursing education and working conditions pays for itself[26]. Furthermore, hospital management and health care workers should pay attention to the emotional needs of their staff and create an environment of mutual respect and understanding among all cadres. Given the contribution of activist unionism and government policies to poor nurses-doctors working relationships, balanced government and hospital management policy are necessary. The restoration of democracy in Nigeria has already substantially reduced activist union activity in the hospital environment, but more proactive measures are still needed in order to maximize the benefits of investments in the health care sector which remains largely in the public sector in Nigeria as in other developing countries.

## Competing interests

The author(s) declare that they have no competing interests.

## Authors' contributions

RIO conceived the study, participated in the design, administration of questionnaires, entered the data and contributed to drafting the manuscript

CAA analyzed the data and contributed to drafting the manuscript

## Pre-publication history

The pre-publication history for this paper can be accessed here:


